# Urban and Rural Disparities in a WeChat-Based Smoking Cessation Intervention among Chinese Smokers

**DOI:** 10.3390/ijerph18136731

**Published:** 2021-06-23

**Authors:** Ting Luo, Mirandy Li, Donna Williams, Jackson Fritz, Stephen Phillippi, Qingzhao Yu, Stephen Kantrow, Liwei Chen, Yongchun Chen, Kaylin Beiter, Tung-Sung Tseng

**Affiliations:** 1 Department of Family Medicine and Public Health, School of Medicine, University of California, San Diego, CA 92122, USA; mli2@lsuhsc.edu (M.L.); kbeite@lsuhsc.edu (K.B.); 2 Behavioral and Community Health Sciences, School of Public Health, Louisiana State University Health Sciences Center-New Orleans, New Orleans, LA 70122, USA; dwilli3@lsuhsc.edu (D.W.); sphill2@lsuhsc.edu (S.P.); ttseng@lsuhsc.edu (T.-S.T.); 3 School of Medicine, Louisiana State University Health Sciences Center-New Orleans, New Orleans, LA 70122, USA; jfritz@lsuhsc.edu (J.F.); skantr@lsuhsc.edu (S.K.); 4 Biostatistics, School of Public Health, Louisiana State University Health Sciences Center-New Orleans, New Orleans, LA 70122, USA; qyu@lsuhsc.edu; 5 Department of Epidemiology, Fielding School of Public Health, University of California Los Angeles, Los Angeles, CA 90095, USA; cliwei86@g.ucla.edu; 6 Department of Clinical Nutrition, Henan Provincial People’s Hospital, Zhengzhou University People’s Hospital, Zhengzhou 450003, China; henancyc@163.com

**Keywords:** tobacco, smoking cessation, WeChat, disparities, urban and rural

## Abstract

Introduction: Tobacco use, which is directly responsible for 10% of total deaths per year globally, remains consistently high, with approximately 20% of the population reporting regular consumption globally. Moreover, health disparities regarding tobacco consumption and smoking cessation are growing between rural and urban populations worldwide. Social media interventions for tobacco cessation may effectively reach both groups. The objective of this study was to evaluate the efficacy of a WeChat-delivered smoking cessation intervention among rural and urban Chinese smokers, and to assess moderating variables that may contribute to differential intervention efficacy. Methods: WeChat was used to recruit smokers into this intervention study between 1 July and 5 August 2019. Participants were randomized to one of three intervention schedules: participants in the Standard Group and the Enhanced Group received 20 smoking-related messages over 2 weeks, whereas participants in the Enhanced Group received an extra 6 oral health-related messages for one week. Participants in the control group received 20 smoking-related messages after the post-intervention assessment. Participants completed questionnaires at baseline and at 4 weeks follow-up. Our primary outcome was smoking cessation stage of change and secondary outcome was 24-h point prevalence abstinence (PPA). Urban and rural areas were based on self-reported living areas. Chi-squared test, Fisher’s exact test, ANOVA test, linear regression, and logistic regression were used for analysis. Results: Overall, 403 participants completed the intervention (233 rural, 107 suburban, 63 urban). Compared to urban participants, rural participants were more likely to have progressed to a later stage of change (β = 0.40, 95% CI: 0.13, 0.67) and to report higher 24-h PPA rates at follow-up (aOR = 3.23, 95% CI: 1.36, 7.68). After stratification by living area, the intervention effects in stage of change and 24-h PPA rate at follow-up were only found in the urban subgroup. Discussion: Smokers who lived in rural areas reported better smoking cessation outcomes compared with urban smokers; however, the efficacy of a WeChat-based smoking cessation intervention was only found for participants living in an urban area. WeChat based smoking cessation interventions should be used to promote smoking cessation in urban, suburban, and rural areas.

## 1. Introduction

Smoking is a leading cause of death and disability worldwide. In 2018, over 1.3 billion people smoked tobacco globally [1], and tobacco accounted for more than 1 in 10 deaths [2]. Approximately 80% of all smokers worldwide live in low- and middle-income countries [2]. China has the largest number of tobacco consumers, and accounts for nearly one-quarter of smokers worldwide [3,4]. According to the 2018 Global Adult Tobacco Survey (GATS), 26.6% of people ages 15 and older self-identified as smokers, including 50.5% of males and 2.1% of females [4]. Within China, the smoking prevalence is consistently higher in rural areas than in urban areas [5,6,7]. The overall smoking prevalence in rural and urban areas was 29.8% and 26.1%, respectively, with a significant difference among males in particular living in rural versus urban areas: 56.1% and 49.2%, respectively [8]. These differences in smoking prevalence by living area indicate a health disparity between rural and urban areas. This disparity in smoking prevalence heavily influences long-term health outcomes: male smokers living in rural areas were more likely to develop cancers than smokers in urban areas [9].

Although recent headway has been made to reduce smoking prevalence in China, more than 300 million Chinese persons aged 15 and older continue to smoke [2], and the smoking cessation rate in China remains low [8]. One-third of smokers attempted to quit during the past 12 months, while 91.8% did not use any cessation methods, despite knowing the efficacy of interventions such as pharmacotherapy medications and counseling [8]. Similarly, Chen et al. also pointed out that less than 10% of Chinese former smokers had quit by choice (as opposed to quitting due to a major illness) [10]. Previous studies indicate that rural China in particular has a lack of smoking cessation services to help smokers quit [5,6,7]. This inaccessibility of cessation services is likely one reason why smoking cessation efforts are low among many smokers.

The differences in smoking prevalence and smoking cessation rates between rural and urban areas may be attributed to both individual factors (e.g., education, income) and systematic factors (e.g., lack of smoking cessation services). Social media-based interventions have the potential to address disparity-associated factors at both levels. Social media provides many functions, such as social network, media sharing networks, discussion forms, blogging and publishing networks, etc., and hence it can be used to connect individuals and build new social networks. Social media-based interventions can also reduce the impact of systemic barriers to cessation services.

WeChat was developed by the Tencent Company in China and was first released on 21 January 2011. WeChat now has become the most utilized and inexpensive social media platform in China, with 1 billion monthly active users [11]. WeChat provides a wide range of functions through a single internet-based platform, including free calls, free messaging, hold-to-talk voice messaging, broadcast (one-to-many) messaging, group messaging, video conferencing, movie tickets, bill payments, appointment making, social networks, taxi orders, investments, and video games, and therefore WeChat has become indispensable. An average WeChat user spends 66 min a day on the platform. About 45 billion messages are sent daily by WeChat users and over 200 million bank cards are linked to WeChat Pay [12]. WeChat is also been widely used in work environments, instead of using email—70% of Chinese workers depend on WeChat for work-related communication as an alternative to email [13]. The peak WeChat usage occurs in the 26–35 age group, although a high proportion of individuals in older age groups also use WeChat [11]. According to the 2020 statistics from CNNIC (China Internet Network Information Center), the number of internet users in Chinese rural areas has significantly increased to 46.2% in March 2020 (vs. 76.5% in Chinese urban areas) compared to 28.1% in 2007 (vs. 60.3% in Chinese urban areas) [14].

The literature has shown that China’s rural areas usually have higher smoking rates and fewer smoking cessation services [4]. The increasing availability of internet connection in rural China has allowed for an extension of WeChat functionality to a larger proportion of the entire population of China [14]. Furthermore, WeChat is slowly changing the methods people use receive information, and people like to receive health education via WeChat. WeChat has been used to aid many health behavior changes and disease prevention and control, such as asthma, diabetes, weight loss, and chronic rhinosinusitis [15]. In particular, WeChat-based health education platforms have proven to be widely accepted in rural China [15]. Thus, despite considering the current existing economic and cultural barriers between rural and urban areas, WeChat remains a potential way to improve smoking cessation in rural China. Specifically, implementing smoking cessation interventions using WeChat may mitigate the lack of smoking cessation services in rural areas. Using WeChat for smoking cessation interventions in urban or rural areas may result in comparable effects. Thus, we hypothesized that there is no difference in the efficacy of a WeChat-based intervention between urban and rural Chinese smokers. However, only few studies have examined the impact of smoking cessation outcomes that target regular smokers according to their living area in urban and rural areas in China. Therefore, the objective of this study was to evaluate the efficacy of a WeChat-delivered smoking cessation intervention among rural and urban Chinese smokers, and to assess moderating variables that may contribute to differential intervention efficacy.

## 2. Methods

### 2.1. Program and Participants Description

The study was a secondary analysis of a randomized controlled trial, which was primarily developed to evaluate the efficacy of a WeChat-based smoking cessation intervention, and to examine a possible additive effect of integrating oral health and smoking-related information into a smoking cessation intervention. We added the oral health condition into the intervention content because tobacco use is one of the major causes of oral disease burden worldwide [16]. Among all factors that contribute to oral diseases, smoking cannot be underestimated [16]. Because quitting smoking can lower the risk of periodontitis and other oral diseases, dental health providers often promote smoking cessation. However, limited studies have examined the effect of promoting oral health awareness on improving smoking cessation outcomes [17]. Study participants were recruited using WeChat from 1 July to 5 August 2019. Recruitment materials were posted and advertised on a WeChat public platform (Chinese Clinical Nutrition Network), WeChat moments, WeChat groups, Weibo (Chinese Twitter, but there are twice as many active users on Weibo as on Twitter), and QQ (the second most popular instant messenger platform). Individuals could “friend” the project account to test their eligibility for this study by answering a few short questions. Inclusion criteria were (a) being a current smoker, (b) currently living in China, (c) being aged 18 years and older, (d) being an active user of WeChat (login at least once a day), and (e) willingness to participate in the study. Current smokers were defined as respondents who smoked 100 or more cigarettes in their lifetime and currently smoke every day or some days [18]. Eligible and consenting participants were randomly assigned to one of three groups: either one of the two intervention groups (smoking cessation only; smoking cessation + oral health education) or the wait-list control group.

Participants in the Standard Group received 20 smoking-related messages (e.g., videos or images) over 2 weeks; participants in the Enhanced Group received the same information as the Standard Group plus an extra 6 oral health-related messages for one week after the other information was given. Participants in the control group received 20 smoking-related messages after the post-intervention assessment. The topics of intervention messages can be found in Table S1.

The transtheoretical model (TTM) has been demonstrated as a guide for smoking cessation, and consequently our intervention content was based on previous literature and the TTM [19]. We included six intervention content categories: consciousness raising, self-efficacy, helping relationship, stimulus control, coping skills, and oral health awareness.

### 2.2. Measures

#### 2.2.1. Baseline and Follow-Up Questionnaires

All the participants were asked to complete questionnaires at baseline and at 4 weeks follow-up. Demographic information was only collected at baseline. Smoking behavior information, TTM theoretical concept measurements, and other health-related indicators were collected at both baseline and at 4 weeks follow-up. All assessment surveys were delivered via “Wenjuanwang”, a popular China marketing research tool [20]. Questionnaires were linked by participants’ unique smoker ID, which was required to be filled in for each iteration of the questionnaire.

#### 2.2.2. Living Areas and Demographic Measures

Living areas were classified as urban, suburban, and rural areas for analysis, which were self-reported by participants at the time when the baseline survey was conducted. “Urban” refers to participants who live in prefecture-level cities or county-level cities, “suburban” refers to participants who live in the areas beyond a city’s border or live in towns, and “rural” refers to participants who live in villages. The following demographic data were collected: age, gender (male or female), household income in RMB (≤49,999, 50,000–99,999, 100,000–199,999, ≥200,000). The annual household income was based on a Chinese government report. The current exchange rate for USD to RMB was as considered as follows: RMB 6.5 = USD 1; RMB < 49,999 = USD 7692; RMB 50,000–99,999 = USD 7692–15,384); RMB 100,000–199,999 = USD 15,384–30,769; RMB >200,000 = USD >30,769. “Hukou” type was also measured and classified as non-agricultural register and agricultural register. “Hukou” is a family registration program that serves as a domestic passport and regulates population distribution and rural-to-urban migration in China. In addition, participants provided their education level (high school or less, association college, college and above), marital status (married, single and other), occupation (business, government/agency officers/professional staff, labor workers, self-employed and other), BMI based on self-reported height and weight (underweight and normal weight BMI < 23, overweight and obese BMI ≥ 23), and age of smoking initiation. Participant age was considered as both a continuous and categorical variable (18–24, 25–29, 30–39, ≥40 years) during statistical analyses. Age of smoking initiation was only considered as a continuous variable.

#### 2.2.3. Smoking Cessation Measures

The primary outcome for this study was smoking cessation stage of change, as defined by the TTM model [21]. A smoker’s stage of readiness before “action” has typically been assessed with the question “When do you intend to quit smoking?” Smokers who responded “do not intend to quit” were categorized as being in the precontemplation stage; those who responded “within next the 6 months” were categorized as being in the contemplation stage; those who responded “within next the 30 days” were categorized as being in the preparation stage. Smokers who responded “No” to the question “Have you smoked any cigarettes in the past 7 days, even a puff?” were categorized as being in the action stage. Smokers in the precontemplation stage were given a score of 1, smokers in the contemplation stage were given a score of 2, smokers in the preparation stage were given a score of 3, and smokers in the action stage were given a score of 4 [22]. Previous studies have shown that in short-term interventions, smokers are more likely to move to an adjacent stage than to a distal one [22]. Our secondary outcome was 24-h point prevalence abstinence (PPA), which was measured by the question “Have you smoked any cigarettes or used other tobacco, even a puff, in the last 24 h?” The change in 24-day PPA rates was classified into two groups: progressed and did not progress. “Progressed” refers to participants that changed their reported 24-day PPA rate from “has smoked in the past 24 h” at baseline to “has not smoked in the past 24 h” at follow-up. “Did not progress” includes those who regressed or had no change. All measures were delivered in Chinese, translated, and verified by a native speaker on the study team prior to intervention implementation.

### 2.3. Analysis

We tested demographic factors and smoking behavior (13 factors in total) comparisons according to participant completion and attrition status (see Table S2). Although we had a relatively high attrition rate (46.4%), the only significant difference was observed in sex (male: 92.6% for follow-up vs. 84.5% for attrition). Participants who completed the follow-up assessment showed similar demographic characteristics and smoking behaviors as those who did not complete the follow-up assessment; thus, the missing data were considered as missing completely at random. In addition, we controlled for gender in the later analysis; therefore, we concluded that the study results were not influenced significantly by attrition.

We conducted an observational study analysis on the basis of the intervention study. We used descriptive statistics to describe demographic factors according to living areas and smoking behaviors by living area. In this study, we set up alpha level at 0.05. We applied chi-squared tests or Fisher’s exact tests to examine differences between baseline and follow-up among groups for categorical outcome variables (e.g., gender, education level). Similarly, we applied ANOVA with Tukey’s pairwise comparison tests to examine intergroup differences within continuous outcome variables (e.g., smoking cessation stage of change).

Linear regressions analysis was used to assess relationships between multiple covariates and stage of change at follow-up. We considered smoking cessation stage of change as a continuous variable. Questionnaire responses were scored with the assumption that stages of change are sequential. Smokers in the pre-contemplation stage were scored as 1, smokers in the contemplation stage were scored as 2, smokers in the preparation stage were scored as 3, and smokers in the action stage were scored as 4. Logistic regressions were used with change in 24-h PPA. In the crude models, we examined simple associations between each outcome and each demographic variable. In the adjusted models, we included the study group and all demographic variables (age, gender, education level, and income level) as covariates. As shown in Table 1, education was significantly different between living areas. Moreover, gender was identified as a significant factor in attrition analysis (between completion and attrition status). In addition, previous literature has demonstrated that smoking cessation effect is greatly influenced by age, gender, education level, and income level. Due to limitations from a small sample size, we did not include other factors which that not been consistently proven to be strongly associated with smoking cessation behaviors. Finally, stratifying analysis was used to examine the moderating effect of self-reported living area (urban/suburban/rural) on the efficacy of using WeChat for smoking cessation. Suburban is a transition between rural and urban. Compared to people who live in urban areas in China, people who live in suburban areas tend to have lower educational levels and a higher level of poverty. In contrast, compared to people who live in rural areas in China, people who live in suburban areas tend to have higher educational levels and lower levels of poverty. Thus, this study also examined the cessation effect in suburban areas.

## 3. Results

### 3.1. Demographic Information and Smoking Behavior at Baseline by Living Area

**Figure 1:**
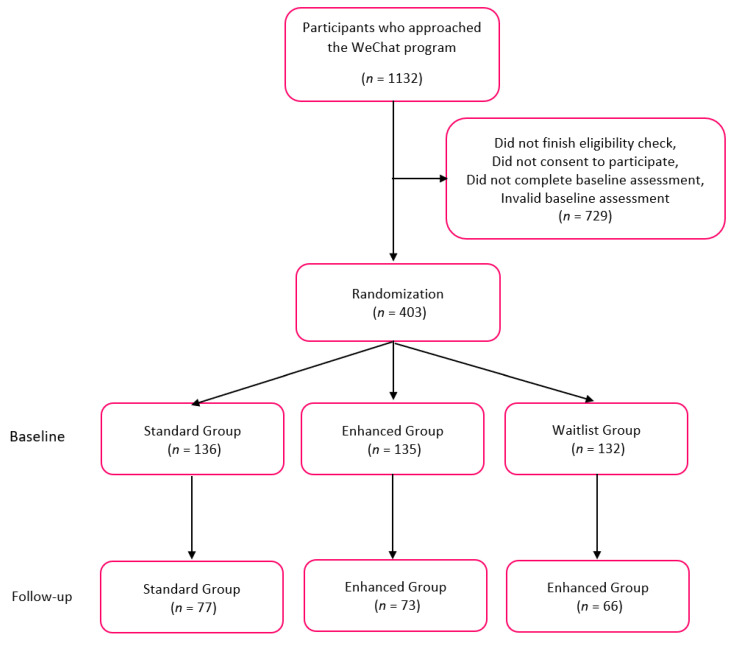
Flow chart of randomization.

At baseline, 403 smokers were eligible and consented to the study. Of these participants, 233 (57.8%) lived in urban areas, 107 (26.6%) lived in suburban areas, and 63 (15.6%) lived in rural areas (see Figure 1 for flow chart). Table 1 shows detailed demographic information and smoking behaviors for participants at baseline by living area. For demographic indicators, significant differences were found between participants who lived in urban, suburban, and rural areas according to age groups, household income, household registration, education level, and occupation. All definitions are included in Table 1. No significant differences were found between participants who lived in urban, suburban, and rural areas for age (as a continuous variable), sex, marital status, BMI, age at smoking initiation, and study group. The mean age overall was 30.5, the mean age at smoking initiation was 18.1, and the mean duration of smoking habit was 12.6 years. The majority of participants (88.8%) were male, 60.1% of participants were married, and 42.4% of participants were overweight and obese. There was a higher proportion of participants who lived in rural areas with a household income RMB ≤ 100,000 compared to the proportion of participants who lived in suburban and urban areas (85.7% vs. 54.9% and 73.9%, respectively). Participants who lived in urban areas were more likely to have a higher education level (college and above), compared to participants who lived in suburban and rural areas (38.6% vs. 22.4% and 11.1%, respectively). Participants who lived in urban areas were more likely to be employed in business or as government/agency officers/professional staff, while participants who lived in rural areas were more likely to be employed as labor workers, self-employed, or in some other occupation. Among those participants who lived in urban areas, 76.8% of them were listed on the non-agricultural registry, compared with 43.0% of suburban participants and 14.3% of rural participants.

For smoking behavior indicators, significant differences in baseline TTM stage of change were found at among participants who lived in urban, suburban, and rural areas for stage of change (TTM): the majority of participants who lived in suburban and rural areas were in the preparation stage; the majority of participants who lived in urban areas were in the contemplation stage. No significant differences were found between participants who lived in urban, suburban, and rural areas for baseline 24-h PPA rates, 7-day PPA rates, attempt to quit, daily cigarette use, and nicotine dependence score at baseline.

### 3.2. The Comparison of Change in Smoking Behaviors between Baseline and Follow-Up for Urban, Suburban, and Rural Participants

Table 2 shows the change in smoking behavior between baseline and follow-up for urban, suburban, and rural participants. Overall, significant differences were found by living areas for stage of change and change in 24-h PPA rates. Participants who lived in rural areas were more likely to progress to a later stage compared with participants who lived in urban areas or suburban areas (44.1% vs. 29.8% and 22.4%, respectively). Similarly, participants who lived in rural areas were more likely to report 24-h PPA compared with participants who lived in urban areas or suburban areas (52.9% vs. 26.6% and 27.6%, respectively).

### 3.3. Associations between Smoking Cessation Outcomes and Living Areas

For overall participants, compared to the participants who lived in urban areas, participants who lived in rural areas were more likely to move to a later stage of quitting and were more likely to report 24-h PPA progression.

### 3.4. Stage of Change

Stage of change was analyzed as a continuous variable with a range 1–4. Table 3 shows the associations between stage of change at follow-up and each risk factor in both crude models and the adjusted model. A statistically significant difference was found in stage of change at follow-up between participants who lived in rural areas and urban areas (β = 0.32, 95% CI: 0.02–0.62), but was not found between participants who lived in suburban areas and urban areas (β = 0.02, 95% CI: −0.23, 0.27). The positive association indicates that participants who lived in rural areas were more likely to report a later stage of change and were more likely to quit. Participants who lived in rural areas were more likely to move towards later stages of quitting, compared to participants who lived in urban or suburban areas. The difference in reported stage of change at follow-up between participants who lived in urban and suburban areas was very small.

After adjusting for all demographic variables and groups, we still observed a statistically significant difference between participants who lived in urban and rural areas: participants who lived in rural areas were more likely to report a later stage of change at follow-up when compared with participants who lived in urban areas (β = 0.35, 95% CI: 0.04–0.67), although this was not significant. Thus, participants who lived in rural areas were also more likely to move to a later stage of quitting from their baseline stage in the adjusted model compared to participants who lived in urban or suburban areas.

### 3.5. 24-h PPA

Change in 24-h PPA rate was classified into two groups: progressed and did not progress. Table 4 shows the association between 24-h PPA rate progression status and each risk factor in crude models and an adjusted model. Significant differences were found in crude models when examining 24-h PPA rate progression with household registration, self-reported living area, marital status, self-efficacy score at follow-up, and coping skills score at follow-up. Participants from the Enhanced Group (vs. control), who were registered as an agricultural household (vs. non-agricultural household), and who were married (vs. single) were more likely to report 24-h PPA rate progression.

Compared to participants who lived in urban areas, participants who lived in rural areas were more likely to report 24-h PPA rate progression (OR = 2.57, 95% CI: 1.15–5.76). No statistically significant differences were found for 24-h PPA rate progression between participants who lived in suburban and urban areas. In contrast, participants who lived in rural areas were more likely to have 24-h PPA rate progression versus those participants who lived in urban or suburban areas. The difference in 24-h PPA rate progression between participants in urban and suburban areas was very small. After adjusting for all demographic indicators, group number, and marital status, we found that significant differences remained between participants in rural and urban areas, with rural participants more likely to report 24-h abstinence progression (aOR = 3.23, 95% CI: 1.36–7.68) compared to urban ones. No significant difference was observed between participants in suburban and urban areas.

### 3.6. Moderating Analysis

An interaction term between study groups and living area was also assessed, but it was found to not be significant (see Table 5 for the *p*-values for the interaction between living area and study group). In order to compare intervention effect by living areas, we conducted a stratified analysis by living area. However, due to the small sample size for rural participants, we decided to only include demographic factors in the adjusted model. Among urban-based participants, those in the Enhanced Group were more likely to move to a later stage of quitting and were more likely to report 24-h PPA progression relative to the wait-list control group. Table 5 shows the associations between group and selected outcomes in the adjusted model for urban, suburban, and rural participants.

### 3.7. Stage of Change

A statistically significant difference was found in stage of change at follow-up for urban participants. More specifically, for urban participants, the Standard Group (beta = 0.33, 95% CI: 0.00–0.66) and the Enhanced Group (beta = 0.66, 95% CI: 0.32–1.00) were more likely to move to a later stage of quitting compared with the Waitlist Group. Participants in the Enhanced Group were most likely to move towards later stages of quitting, followed by the Standard Group and the Waitlist Group. Additionally, urban males were more likely to move towards later stages of quitting than urban females. No differences in TTM stage movement were found among rural or suburban participants.

### 3.8. 24-h PPA

A statistically significant change from baseline to 4 weeks follow-up was also found in 24-h PPA rate for urban participants, but not among suburban or rural participants. Urban participants in the Enhanced Group were statistically more likely to report 24-h PPA progression, compared to the Waitlist Group (OR = 4.19, 95% CI: 1.27–13.78). However, although a statistically significant difference was not found between the Standard Group and the Waitlist Group for urban participants, urban participants in the Standard Group were more likely to report 24-h PPA progression compared to the Waitlist Group (OR = 1.56, 95% CI: 0.46–5.23).

## 4. Discussion

Participants living in rural areas were more likely to report better smoking cessation outcomes versus urban participants. Suburban participants were more similar to urban than rural. Below, we discuss the urban and rural disparities we observed, as well as the impact of our WeChat intervention in these groups.

### 4.1. Urban and Rural Disparities

Compared to participants in urban or suburban areas, participants in rural areas were more likely to progress to a later stage (29.8%, 22.4%, vs. 44.1%, respectively) and report higher 24-h PPA rates at follow-up (30.6%, 31.0%, vs. 52.9%, respectively). To our knowledge, few intervention studies have compared the smoking cessation outcomes between urban and rural populations in China, especially using social media as an intervention tool. Our study perhaps is one of the first studies to compare the difference in smoking cessation outcomes for urban and rural participants. However, compared to studies of urban/rural smoking cessation conducted in the United States or in Canada, our findings were in contradiction with those studies [23,24,25]. Previous studies have suggested that smokers living in rural areas are less likely to quit than smokers who lived in urban areas [23,24,25]. More specifically, Carlson et al. found that continuous abstinence rates were 27.5% in urban sites and 25.5% in rural sites after a telehealth-delivered intervention completion in Canada [23]. Similarly, Northridge et al. also found that rural residents reported a lower end-of-class quit success rate than those urban residents (aOR = 0.58, 95% CI = 0.35, 0.94) in the United States [25]. Although smoking cessation outcomes are not the same, we still can see a trend that smokers who lived in urban areas reported better smoking cessation outcomes compared to smokers who lived in rural areas.

Cultural factors related to smoking and the rural–urban divide in China may have contributed to this reversal of Western data. The increased efficacy of our intervention among rural participants relative to urban participants may be related to the fact that rural smokers in general have lower access to cessation services at baseline. Mao et al. found that living in rural areas was a barrier to quitting smoking because of a lack of information on smoking cessation [7]. More services, including those building on our pilot intervention program, should be expanded and implemented in rural areas in order to decease this disparity. Alternatively, our findings may be related to a higher severity of smoking and nicotine dependence among rural smokers. The World Health Organization has demonstrated that the smoking prevalence in China was higher in rural areas than in urban areas [8,26]. In 2019, Lee et al. found that compared to rural participants, urban participants smoked fewer cigarettes per day (IRR (incidence rate ratio) = 0.93, 95% CI (0.89, 0.98)) and were less likely to report being current smokers (aOR = 0.83, 95% CI (0.74, 0.95)) [6]. In the same year, Zhao et al. also reported that rural residents were more likely to smoke in the city vs. surrounding areas of Qingdao in China [5]. Mao et al. found that lifestyle differences among rural vs. urban residents may affect smoking behaviors: rural residents may be influenced by courtyard-based leisure activities that facilitated smoking [7]. Thus, the increased efficacy of this intervention among rural smokers may be confounded by smoking behaviors. Nevertheless, after stratifying by living area, the intervention effects in stage of change at follow-up and 24-h PPA rate were only found among participants in urban areas, indicating intervention effects primarily come from urban participants. The intervention effects were not observed among rural participants perhaps due limited sample size for rural participants. A study with enough power is required to confirm these results.

### 4.2. Using WeChat to Mitigate the Difference between Urban and Rural

WeChat has become one of the most inexpensive and widely utilized social media platforms in China, with both rural and urban users [27]. Implementing smoking cessation interventions using WeChat may thus mitigate the lack of smoking cessation services in rural areas. Moreover, acceptance of social media-based interventions for tobacco cessation is high among rural smokers [28]. Smokers who lived in rural areas had better smoking cessation outcomes; however, the effectiveness of a WeChat-based intervention was only found in the urban area. This finding may result from the smaller sample size of participants from the rural area. WeChat should be expanded for interventions in this capacity to support rural health.

### 4.3. Using WeChat to Aid Smoking Cessation

One of the most important functional modules of WeChat is “WeChat official accounts”, which is the equivalent of a Facebook page, and it can be used for organizations, communities, celebrities, developers, or companies [29]. WeChat official accounts can be used to gather followers, push notifications, and redirect users to a website/e-commerce [29]. About 20 million active WeChat official accounts existed as of 2020, and many WeChat China users consider access of the platform as their main information source [13]. Over 50% of WeChat users spend more than a half an hour per day following official accounts [13]. Therefore, WeChat official accounts may play a key role in future smoking cessation programs, which can either create new official account or collaborate with existing official accounts. Cessation programs can also cooperate with local health department, hospitals, schools, or communities to target different groups of people.

Additionally, clinical group counseling has a long history of being able to help smokers quit smoking. Many factors influence attendance rates, such as distance between home and class site or availability of transportation [30]. As a result, it is not easily scaled up to provide continuous help to large numbers of people who smoke, which usually requires smokers to physically attend class [31]. Future studies incorporating WeChat and clinical group counseling programs may be able to share resources and provide a comprehensive, multi-faceted approach to smoking cessation.

### 4.4. Limitations and Strengths

This analysis was based on a RCT smoking cessation intervention; however, we did not stratify participant recruitment by living area. Thus, we only had 34 and 58 smokers who lived in rural or suburban areas included in this analysis. Due to the sample size limitation, warnings occurred during logistic regression analysis and CIs had a wide range when we run subgroups analysis, the results should be interpreted with caution. Thus, we are not able to confidently summarize that rural residents were more likely to progress to a later stage and had a higher 24-h PPA rate. To confirm the higher smoking cessation rates using WeChat for participants who lived in rural areas compared to participants who lived in urban areas, we require a RCT study with a larger sample size, especially in terms of recruitment by living area. Moreover, our sampling methods were convenience and snowball sampling, which are non-probability selection methods. Therefore, the results of the sample may not be generalizable to all WeChat users, nor all Chinese smokers. Furthermore, our measurements are based on self-reported data, without bio-chemical validation. Self-reported data may be exaggerated, as participants may have been embarrassed to report that they were unsuccessful in their quitting behaviors. Finally, this study did not consider rural–urban migration. Many people, especially young people, were born in rural areas, but later worked in urban areas. Although they reported living in rural areas, they may not have the same benefits as urban residency, since they did not have a city household registration, also called Hukou.

Despite these limitations, this study remains an important, novel evaluation of a smoking cessation intervention and provides implications for addressing a large health disparity. We found that smokers who lived in rural areas were more likely to progressed to later stages and had higher 24-h PPA rate than participants who lived in urban areas in the adjusted model. Few studies have examined the efficacy of smoking cessation interventions targeted to the rural population in China, and fewer have explicitly evaluated outcomes for such interventions between urban and rural populations in China. Our study perhaps is one of the few studies to compare the difference in smoking cessation outcomes for urban and rural participants. Additionally, the study proved that a WeChat-based intervention is more likely to be effective in rural areas in terms of effective analysis, which may help mitigate urban and rural smoking cessation disparities.

## 5. Conclusions

Smokers who lived in rural areas had better smoking cessation outcomes compared with urban smokers; however, the efficacy of a WeChat-based smoking cessation intervention was only found in an urban area. WeChat-based smoking cessation interventions have the potential to reach one-quarter of smokers worldwide, especially smokers from rural areas in China, where smoking is considered a symbol of maturity and independence. Consequently, WeChat-based smoking cessation intervention should be used to promote smoking cessation in urban, suburban, and rural areas. In addition, social factors contribute to an individual’s decision to smoke in China. Thus, there are many socio-cultural factors that hinder the efficacy of standard smoking cessation interventions; therefore, future culturally relevant interventions with large sample sizes are needed.

## Figures and Tables

**Table 1:** Demographic information at baseline by living area.

Variables		Urban (*n* = 124)		Suburban (*n* = 58)		Rural (*n* = 34)		*p*-Value
		*n*	%	*n*	%	*n*	%	
Group								0.36
	Standard group	46	37.1	23	39.7	8	23.5	
	Enhanced group	42	33.9	20	34.5	11	32.4	
	Waitlist group	36	29.0	15	25.9	15	44.1	
Demographic Information								
Age (Mean, SD)		30.3	9.3	27.8	6.5	28.1	8.5	0.14
Age Category								0.13
	18–24	37	30.1	21	36.8	15	45.5	
	25–29	37	30.1	14	24.6	7	21.2	
	30–39	27	22.0	19	33.3	8	24.2	
	≥40	22	17.9	3	5.3	3	9.1	
Sex								0.16
	Male	112	90.3	54	93.1	34	100.0	
	Female	12	9.7	4	6.9	0	0.0	
Household Income ^1^ in RMB								0.21
	≤49,999	37	29.8	21	36.1	13	38.2	
	50,000–99,999	33	26.6	20	34.5	14	41.2	
	100,000–199,999	38	30.7	10	17.2	5	14.7	
	≥200,000	16	12.9	7	12.2	2	5.9	
Hukou Type ^2^ (Household Registration)								<0.01
	Non-agricultural register ^3^	99	79.8	23	40.0	4	11.8	
	Agricultural register^3^	25	20.2	35	60.3	30	88.2	
Education Level								<0.01
	High school or less	33	26.1	23	39.7	21	61.8	
	Associate college	46	37.1	22	37.9	9	26.5	
	College and above	45	36.3	13	22.4	4	11.8	
Marital Status								0.92
	Married	73	58.9	35	60.3	19	55.9	
	Single ^4^	51	41.1	23	39.7	15	44.1	
Occupation								0.02
	Business ^5^	68	54.8	21	36.2	11	32.4	
	Government/agency officers/professional staff ^6^	25	20.2	11	19.0	4	11.8	
	Labor Workers ^7^	11	8.9	10	17.2	7	20.6	
	Self-employed and other ^8^	20	16.1	16	27.6	12	35.3	
BMI ^9^								<0.01
	Underweight and normal weight	70	56.9	46	79.3	14	41.2	
	Overweight and obese	53	43.1	12	20.7	20	58.8	
Smoking Behavior								
Age at Smoking Initiation (mean, SD)		17.8	3.8	18.7	4.3	18.1	3.9	0.35
Duration of Smoking Habit (mean, SD)		12.6	9.0	9.1	6.0	10.1	7.2	0.02
Stage of Change								0.03
	Pre-contemplation	8	6.5	2	3.5	5	14.7	
	Contemplation	67	54.0	22	37.9	12	35.3	
	Preparation	49	39.5	34	58.6	17	50.0	
Smoked in the Past 24 Hours								0.38
	Yes	116	94.0	54	93.1	34	100	
	No	8	6.5	4	6.9	0	0	
Smoked in the Past 7 Days								
	Yes	124	100	58	100	34	100	
	No	0	0	0	0	0	0	
Attempts to Quit								1.00
	Yes	116	93.6	55	94.8	32	94.1	
	No	8	6.5	3	5.2	2	5.9	
Daily Cigarettes Use								0.44
	10 or fewer	57	46.0	25	43.1	13	38.2	
	11–20	56	45.2	25	43.1	15	44.1	
	21–30	7	5.7	6	10.3	6	17.7	
	31 or more	4	3.2	2	3.4	0	0.0	
Nicotine Dependence (Mean, SD)		4.9	2.4	5.4	2.5	5.5	2.3	0.36

^1^ The current exchange rate for USD to RMB is as follows: RMB 6.5 = USD 1; RMB < 20,000 =< USD 3077; RMB 20,000–49,999 = USD 3077–7692; RMB 50,000–99,999 = USD 7692–15,384; RMB 100,000–199,999 = USD 15,384–30,769; RMB >200,000 = > USD 30,769. ^2^ “Hukou” is a family registration program that serves as a domestic passport, regulating population distribution and rural-to-urban migration. The system is used to determine eligibility for welfare benefits, such as housing, employment, education, and healthcare. ^3^ “Non-agricultural register^”^ refers to the urban household register, and “Agriculture register” refers to the rural household register. ^4^ “Single and other” includes never married, widowed, divorced, and living with partner. ^5^ “Business” includes managers, general office staff, and business service workers (e.g., salesmen, shop clerks, waiters, etc.). ^6^ “Professional staff” includes doctors, teachers, lawyers, journalists, etc. ^7^ “Labor workers” includes factory workers, and farmers/foresters/fishermen. ^8^ “Self-employed” includes self-employed individuals, freelancers, retired individuals, unemployed individuals, students, and others. ^9^ Asian BMI standards are as follows: underweight and normal weight (BMI ≤ 22.9), overweight and obese (BMI ≥ 23).

**Table 2:** Change in smoking cessation outcomes between baseline and follow-up (comparing participants who lived in urban, suburban, and rural areas).

Variables		Urban (*n* = 124)		Suburban (*n* = 58)		Rural (*n* = 34)	
		*n*	%	*n*	%	*n*	%
Stage of Change (Overall *p* = 0.02, χ^2^ = 12.17)							
	Progressed	37	29.8	13	22.4	15	44.1
	Regressed	17	13.7	16	27.6	9	26.5
	No change	70	56.5	29	50.0	10	29.4
Change in 24-h PPA Rates (Overall *p* = 0.12, χ^2^ = 10.45, Table (*p*) using Fisher’s Exact Test < 0.0001)							
	Progressed	33	26.6	16	27.6	18	52.9
	Regressed	3	2.4	2	3.5	0	0
	No change (smoking)	83	66.9	38	65.5	16	47.1
	No change (non-smoking)	5	4.0	2	3.45	0	0

**Table 3:** Association between stage of change at follow-up and risk factors.

Variables		Crude Model			Adjusted Model		
		Beta	95% CI	*p*-Value	Beta	95% CI	*p*-Value
Group							
	Group 1 (standard group)	0.24	(−0.02, 0.50)	0.07	0.23	(−0.03, 0.50)	0.09
	Group 2 (enhanced group)	0.39	(0.13, 0.65)	<0.01	0.40	(0.13, 0.67)	0.004
	Group 3 (waitlist group)	Ref	Ref		Ref	Ref	
Age							
	18–29	Ref	Ref		Ref	Ref	
	≥30	−0.21	(−0.43, −0.02)	0.06	−0.21	(−0.43, 0.01)	0.07
Gender							
	Male	0.33	(−0.07, 0.73)	0.11	0.33	(−0.07, 0.73)	0.10
	Female	Ref	Ref		Ref	Ref	
Household Income							
	≤99,999	Ref	Ref		Ref	Ref	
	≥100,000	0.14	(−0.08, 0.37)	0.20	0.18	(−0.07, 0.42)	0.15
Education Level							
	High school or less	Ref	Ref		Ref	Ref	
	Associate college and above	0.11	(−0.27, 0.18)	0.68	−0.04	(−0.29, 0.20)	0.72
Self-Reported Living Area							
	Urban	Ref	Ref		Ref	Ref	
	Suburban	0.02	(−0.23, 0.27)	0.89	0.02	(−0.23, 0.27)	0.88
	Rural	0.32	(0.02, 0.62)	0.04	0.35	(0.04, 0.67)	0.02
Hukou Type (Household Registration)							
	Non-agricultural register	Ref	Ref		-	-	-
	Agricultural register	0.17	(−0.04, 0.39)	0.11	-	-	-
Marital Status							
	Married	Ref	Ref		-	-	-
	Single and other	0.10	(−0.12, 0.31)	0.39	-	-	-
Occupation				0.93			
	Business	Ref	Ref		-	-	-
	Government/agency officers/professional staff	−0.10	(−0.39, 0.20)	0.51	-	-	-
	Labor workers	−0.04	(−0.38, 0.29)	0.80	-	-	-
	Self-employed and other	0.03	(−0.31, 0.25)	0.82	-	-	-
BMI							
	Underweight or normal weight	Ref	Ref		-	-	-
	Overweight and obese	−0.07	(−0.29, 0.15)	0.51	-	-	-
Age of Smoking Initiation		−0.02	(−0.05, 0.00)	0.06	-	-	-
Nicotine Dependence Score		−0.02	(−0.07, 0.02)	0.33	-	-	-
Baseline Stage		0.25	(0.08, 0.42)	0.004			

Positive association indicates that the intervention group is more likely to report later stage of change and is more likely to quit. Adjusted for group, age, gender, household income, education level, and self-reported living areas.

**Table 4:** Association between change in 24 h PPA rate ^1^ and risk factors.

Variables		Crude Model			Adjusted Model		
		OR	95% CI	*p*-Value	OR	95% CI	*p*-Value
Group							
	Group 1 (standard group)	1.45	(0.68, 3.08)	0.34	1.64	(0.73, 3.67)	0.95
	Group 2 (enhanced group)	2.24	(1.07, 4.71)	0.03	2.57	(1.15, 5.76)	0.03
	Group 3 (waitlist group)	Ref	Ref		Ref	Ref	
Age							
	18–29	Ref	Ref		Ref	Ref	
	≥30	0.63	(0.35, 1.17)	0.22	0.67	(0.35, 1.28)	0.22
Gender							
	Male	2.04	(0.56, 7.41)	0.28	2.00	(0.53, 7.57)	0.31
	Female	Ref	Ref		Ref	Ref	
Household Income							
	≤99,999	Ref	Ref		Ref	Ref	
	≥100,000	0.98	(0.54, 1.79)	0.95	1.18	(0.58, 2.41)	0.64
Education Level							
	High school or less	Ref	Ref		Ref	Ref	
	Associate college or above	0.69	(0.38, 1.26)	0.23	0.81	(0.40, 1.63)	0.55
Self-reported Living Area							
	Urban	Ref	Ref		Ref	Ref	
	Suburban	1.05	(0.52, 2.11)	0.89	1.03	(0.49, 2.13)	0.13
	Rural	3.10	(1.42, 6.78)	<0.01	3.23	(1.36, 7.68)	0.006
Hukou Type (Household Registration)							
	Non-agricultural register	Ref	Ref		-	-	-
	Agricultural register	2.04	(1.14, 3.67)	0.02	-	-	-
Marital Status							
	Married	Ref	Ref		-	-	-
	Single and other	1.92	(1.07, 3.45)	0.03	-	-	-
Occupation							
	Business	Ref	Ref		-	-	-
	Government/agency officers/professional staff	0.95	(0.43, 2.12)	0.91	-	-	-
	Labor workers	0.89	(0.35, 2.24)	0.81	-	-	-
	Self-employed and other	1.11	(0.53, 2.32)	0.78	-	-	-
BMI							
	Underweight and normal weight	Ref	Ref		-	-	-
	Overweight and obese	0.87	(0.48, 1.58)	0.65	-	-	-
Age of Smoking Initiation		1.04	(0.96, 1.12)	0.33	-	-	-
Nicotine Dependence Score		0.95	(0.85, 1.07)	0.43			
Baseline Stage		1.10	(0.69, 1.76)	0.70			

Adjusted for group, age, gender, household income, education level, and self-reported living ar-eas.

**Table 5:** Associations between group and selected outcomes in adjusted model for rural, suburban, and rural participants.

Variables		Adjusted Model ^1^		
		Beta	95% CI	*p*-Value
Stage of Change at Follow-up Crude Model: Living Area * Group (*p* = 0.48) ^2^ Adjusted Model: Living Area * Group (*p* = 0.47)				
Stage of Change at Follow-up for Urban Participants				
	Standard group	0.33	(0.00, 0.66)	0.05
	Enhanced group	0.66	(0.32, 1.00)	<0.001
	Waitlist group	Ref	Ref	
Stage of Change at Follow-up for Suburban Participants				
	Standard group	−0.04	(−0.58, 0.50)	0.88
	Enhanced group	0.20	(−0.54, 0.58)	0.94
	Waitlist group	Ref	Ref	
Stage of Change at Follow-up for Rural Participants				
	Standard group	0.24	(−0.62, 1.10)	0.57
	Enhanced group	0.11	(−0.73, 0.96)	0.79
	Waitlist group	Ref	Ref	
Change in 24-h PPA Rate Crude Model: Living Area * Group (*p* = 0.39) ^2^ Adjusted Model: Living Area * Group (*p* = 0.31)				
		OR	95% CI	*p*-value
Change in 24-h PPA Rate for Urban Participants				
	Standard group	1.56	(0.46, 5.23)	0.56
	Enhanced group	4.19	(1.27, 13.78)	0.009
	Waitlist group	Ref	Ref	
Change in 24-h PPA Rate for Suburban Participants ¤				
	Standard group	1.42	(0.28, 7.23)	0.89
	Enhanced group	1.70	(0.33, 8.67)	0.59
	Waitlist group	Ref	Ref	
Change in 24-h PPA Rate for Rural Participants ¤				
	Standard group	3.37	(0.46, 24.43)	0.15
	Enhanced group	0.68	(0.12, 3.97)	0.27
	Waitlist group	Ref	Ref	

^1^ Adjusted for group, age, gender, household income, and education level. ^2^ Adjusted for group, age, gender, household income, education level, and self-reported living areas. ¤ Warnings occurred during logistic regression analysis. WARNING: There is possibly a quasi-complete separation of data points. The maximum likelihood estimate may not exist. WARNING: The LOGISTIC procedure continues in spite of the above warning. Results shown are based on the last maximum likelihood iteration. Validity of the model fit is questionable. Sex was not available as a control variable due to the small sample size for the female group. * Interaction.

## Data Availability

The data presented in this study are available on request from the corresponding author.

## Supplementary Materials

The following are available online at https://www.mdpi.com/article/10.3390/ijerph18136731/s1, Table S1: Topics of Intervention Messages, Table S2: Demographic Information and Smoking Behaviors at Baseline Comparison between Completion and Attrition.
