# Supplementary material for: Urban and Rural Disparities in a WeChat-Based Smoking Cessation Intervention among Chinese Smokers
Source: Int J Environ Res Public Health. 2021 Jun 23;18(13):6731. doi: 10.3390/ijerph18136731 (PMC8268404; doi:10.3390/ijerph18136731)
Supplement: Supplementary file 1 [file ijerph-18-06731-s001.zip › ijerph-1237784-supplementary.pdf]

Table S1. Topics of Intervention Messages.

Time posted	Number	Topic	Delivered group
		**Intervention Wk1**	
**Wk1day1**	1-2	Harms of smoking & Benefits of quitting smoking	Group 1/2
**Wk1day2**	3-4	Reasons to quit & What is stopping you from quitting smoking?	Group 1/2
**Wk1day3**	5-6	Understanding of nicotine & smoking cessation medications	Group 1/2
**Wk1day4**	7-8	Success stories from Jackie Chan & Success stories from Peiwei Ni	Group 1/2
**Wk1day5**	9-10	How to make a quit plan & self-management approaches	Group 1/2
		**Intervention Wk2**	
**Wk2day1**	11-12	Setting up the social support you need & Seeking social support from us	Group 1/2
**Wk2day2**	13-14	Identifying common triggers and techniques & Saying no to people who hand you cigarettes	Group 1/2
**Wk2day3**	15-16	Coping with withdrawal symptoms & Preventing slips and relapse	Group 1/2
**Wk2day4**	17-18	Physical relaxation techniques & Imaginary relaxation techniques	Group 1/2
**Wk2day5**	19-10	Weight management in diet & in exercise	Group 1/2
		**Intervention Wk 3**	
**Wk3day1**	21-22	Smoking and oral health & Oral health problems	Group 2
**Wk3day2**	23-24	How to take care of teeth & Benefits of receiving routine teeth cleanings and dental checks	Group 2
**Wk3day3**	25-26	The necessity of ultrasonic teeth cleaning & common questions about teeth cleaning	Group 2
		**Intervention for Waitlist Group Wk1**	
**Wk1day1**	1-2	Harms of smoking & Benefits of quitting smoking	Group 3
**Wk1day2**	3-4	Reasons to quit & What is stopping you from quitting smoking?	Group 3
**Wk1day3**	5-6	Understanding of nicotine & smoking cessation medications	Group 3
**Wk1day4**	7-8	Success stories from Jackie Chan & Success stories from Peiwei Ni	Group 3
**Wk1day5**	9-10	How to make a quit plan & self-management approaches	Group 3
		**Intervention for Waitlist Group Wk2**	
**Wk2day1**	11-12	Setting up the social support you need & Seeking social support from us	Group 3
**Wk2day2**	13-14	Identifying common triggers and techniques & Saying no to people who hand you cigarettes	Group 3
**Wk2day3**	15-16	Coping with withdrawal symptoms & Preventing slips and relapse	Group 3
**Wk2day4**	17-18	Physical relaxation techniques & Imaginary relaxation techniques	Group 3
**Wk2day5**	19-10	Weight management in diet & in exercise	Group 3

Group 1: Standard Group, Group 2: Enhanced Group, Group 3: Waitlist Group.

Table S2: Demographic Information and Smoking Behaviors at Baseline Comparison between Completion and Attrition

Variables	Follow-up Completion (n=216)		Follow-up Attrition (n=187)		p-value
	n	%	n	%	
**Age Category**					0.13
18-24	73	34.2	48	26.8	
25-29	58	27.2	42	23.5	
30-39	54	25.4	54	30.2	
≥40	28	13.2	35	19.6	
**Sex**					0.01
Male	200	92.6	158	84.5	
Female	16	7.4	29	15.5	
**Household Income Annually in ¥**					0.52
<50,000	71	32.9	74	39.6	
50,000-99,999	67	31.0	49	26.2	
100,000-199,999	53	24.5	42	22.5	
≥200,000	25	11.6	22	11.8	
**Self-Reported Living Area**					0.98
Urban	124	57.4	109	58.3	
Suburban	58	26.9	49	26.2	
Rural	34	15.7	29	15.5	
**Education Level**					0.24
High School or Less	77	35.7	76	40.6	
Associated College	77	35.7	52	27.8	
College and Above	62	28.7	59	31.6	
**Marital Status**					0.58
Married	127	58.8	115	61.5	
Single	89	41.2	72	38.5	
**Occupation**					0.96
Business	100	46.3	81	43.3	
Government/agency officers/ professional staff	40	18.5	35	18.7	
Labor Workers	28	13.0	26	13.9	
Self-Employed and Other	48	22.2	45	24.1	
**BMI**[h]					0.21
Under-weight and Normal Weight	130	60.5	101	54.3	
Overweight and Obese	85	39.5	85	45.7	
**Age of Smoking Initiation (Mean, SD)**	18.1	3.9	18.1	4.2	0.93
**Stage of change**					0.56
Pre-Contemplation	15	6.9	9	4.8	
Contemplation	101	46.8	84	44.9	
Preparation	100	46.3	94	50.3	
**Smoked in the past 24 hours**					0.74
Yes	204	94.4	178	95.2	
No	12	5.6	9	4.8	
**Smoked in the past 7 days**					
Yes	216	100	187	100	
No	0	0	0	0	

**Daily Cigarettes Use**					0.09
10 or Less	95	44.0	67	35.8	
11-20	96	44.4	83	44.4	
21-30	19	8.8	25	13.4	
31 or More	6	2.8	12	6.4	
**Nicotine Dependence (Mean, SD)**	5.1	2.4	5.4	2.5	0.35
